# Polymorphisms in autophagy genes are genetic susceptibility factors in glioblastoma development

**DOI:** 10.1186/s12885-022-09214-y

**Published:** 2022-02-05

**Authors:** E. Bueno-Martínez, M. Lara-Almunia, C. Rodríguez-Arias, A. Otero-Rodríguez, S. Garfias-Arjona, R. González-Sarmiento

**Affiliations:** 1 Molecular Medicine Unit, Department of Medicine, Institute for Biomedical Research of Salamanca (IBSAL), University of Salamanca, University Hospital of Salamanca, Salamanca, Spain; 2 Neurosurgery Service, Son Espases University Hospital, Mallorca, Spain; 3 Neurosurgery Service, University Hospital of Valladolid, Valladolid, Spain; 4 Neurosurgery Service, University Hospital of Salamanca, Salamanca, Spain; 5 Molecular Medicine Unit, Department of Medicine, Institute for Biomedical Research of Salamanca (IBSAL), Institute of Cancer Molecular and Cellular Biology (IBMCC), University of Salamanca, University of Salamanca-CSIC, 37007 Salamanca, Spain

**Keywords:** Autophagy, Glioblastoma, Polymorphisms, *ATG2B*, *ATG10*, *ATG16L1*, *ATG5*, *NOD2*

## Abstract

**Background:**

Glioblastoma is the most aggressive and common malignant primary brain tumor in adults. Many genetic, epigenetic and genomic mutations have been identified in this tumor, but no driving cause has been identified yet for glioblastoma pathogenesis. Autophagy has proved to be deregulated in different diseases such as cancer where it has a dual role, acting as a tumor suppression mechanism during the first steps of tumor development and promoting cancer cells survival in stablished tumors.

**Methods:**

Here, we aimed to assess the potential association between several candidate polymorphisms in autophagy genes (*ATG2B* rs3759601, *ATG16L1* rs2241880, *ATG10* rs1864183, *ATG5* rs2245214, *NOD2* rs2066844 and rs2066845) and glioblastoma susceptibility.

**Results:**

Our results showed a significant correlation between *ATG2B* rs3759601, *ATG10* rs1864183 and *NOD2* rs2066844 variants and higher risk to suffer glioblastoma. In addition, the relationship between the different clinical features listed in glioblastoma patients and candidate gene polymorphisms was also investigated, finding that *ATG10* rs1864183 might be a promising prognosis factor for this tumor.

**Conclusions:**

This is the first report evaluating the role of different variants in autophagy genes in modulating glioblastoma risk and our results emphasize the importance of autophagy in glioblastoma development.

**Supplementary Information:**

The online version contains supplementary material available at 10.1186/s12885-022-09214-y.

## Background

Glioblastoma (GBM) is the most aggressive and common malignant primary brain tumor in adults [1]. It is a remarkably heterogeneous WHO IV grade glioma characterized by uncontrolled cellular proliferation, strong infiltration capacity, high resistance to apoptosis, intense vascularization, tendency to necrosis and high genomic instability [2]. Despite its diffuse infiltration, GBM rarely metastasizes [3]. The current treatment approach for glioblastoma patients consists of surgical resection when possible, followed by radiotherapy and concomitant and adjuvant temozolomide (TMZ) [4, 5]. Regardless of recent developments in diagnosis and new therapies, prognosis of glioblastoma patients remains very poor, with a survival time of 12–15 months after diagnosis and only about 12% of long-term survivors (more than 36 months) [6–8]. Glioblastoma is 1.6 times more common in men than women and its incidence has been estimated as 2 times higher in Caucasians than in Black populations [9, 10]. Many genetic, epigenetic and genomic modifications have been identified in glioblastoma showing a very complex tumor genetic profile with three commonly activated key signaling pathways (P53 pathway, RB pathway and the receptor tyrosine kinase/Ras/PI3K signaling pathway) and several distinguished gene expression patterns [10, 11]. Nevertheless, no driving cause has been identified for glioblastoma pathogenesis [12]. Several environmental factors have been uncertainly identified as risks, although only high-dose ionizing radiation has showed association to GBM development beyond question [9, 13]. In addition, some inherited genetic syndromes, including Li-Fraumeni syndrome, neurofibromatosis, tuberous sclerosis, retinoblastoma and Turcot’s syndrome, have been demonstrated to increase glioblastoma risk [14]. All this data suggests that glioblastoma predisposition is determined by a complex combination of genetic and still unknown environmental factors [15].

Macroautophagy, commonly referred simply as autophagy, is a highly conserved eukaryotic catalytic mechanism responsible for recycling long-lived, misfolded and aggregated proteins as well as damaged cytoplasmic organelles [16, 17]. This process involves a double-membrane structure called autophagosome that engulfs target molecules to be recycled and fuses with a lysosome. The hydrolases within this organelle will be responsible for the cargo degradation into breakdown products that will be exported back into the cytoplasm to be reused [18, 19]. Autophagy takes place constitutively as a mechanism to maintain cellular homeostasis. However, it is stimulated as a stress response under various conditions including nutrient starvation, acidosis and hypoxia [18, 20]. Additionally, autophagy has proved to be deregulated in different diseases such as type II diabetes, cardiomyopathy, neurodegenerative diseases and cancer. The role of autophagy in cancer appears to be dual, acting as a tumor suppression mechanism during the first steps of tumor development and promoting cancer cell survival in stablished tumors. Therefore, autophagy has been recently identified as a target for therapeutic intervention in cancer, including glioblastoma [7, 17].

In this study, we have analyzed common polymorphisms in genes involved in autophagy (*ATG2B* rs3759601, *ATG16L1* rs2241880, *ATG10* rs1864183, *ATG5* rs2245214, *NOD2* rs2066844 and rs2066845) in order to evaluate their role in the susceptibility to suffer glioblastoma. Furthermore, we have analyzed the distribution of these polymorphisms according to several clinical features listed in patients to find potential biomarkers involved in glioblastoma risk and prognosis.

## Methods

### Study population

A total of 174 Spanish subjects (53.6% males) were included in this association study. The inclusion criteria were newly diagnosed patients with primary glioblastoma according to the 2016 WHO classification [21]. They were recruited in three different Spanish hospitals (University Hospital of Salamanca, University Hospital of Valladolid and Son Espases University Hospital) from 2001 to 2015 and monitored from diagnosis to the present at the Neurosurgery and Medical Oncology Departments in the aforementioned Hospitals. Socio-demographic and clinical data were collected from each patient including variables such as gender, age of diagnosis, tumor location, treatment regimen, surgical resection and disease-free survival. All data were treated with the security measures establish in compliance with the Protection of Personal Data Organic Law 15/1999, 13th December. As control group, 264 sex-matched healthy individuals without personal or familial history of cancer were recruited in the University Hospital of Salamanca. All patients and control subjects signed a written informed consent to participate in this project and the study was approved by the local Ethics Committees of University Hospital of Salamanca, University Hospital of Valladolid and Son Espases University Hospital.

### DNA isolation and SNPs selection and genotyping

Genomic DNA was extracted from leukocytes of patient peripheral blood samples by standard phenol–chloroform procedure. Six polymorphisms in five genes involved in autophagy (*ATG2B* rs3759601, *ATG16L1* rs2241880, *ATG10* rs1864183, *ATG5* rs2245214, *NOD2* rs2066844 and rs2066845) (Table 1) were selected using NCBI SNP database according to the following criteria: > 5% minor allele frequency in Caucasian population, previously described association with disease susceptibility and evidence of functionality. Allelic discrimination assays to genotype the selected polymorphisms were performed using TaqMan® probes (Applied biosystems), with specific oligonucleotides to amplify the polymorphic sequences and two labelled probes with the fluorochrome VIC and FAM to detect both alleles of each polymorphism [22]. PCR reactions were carried out using TaqMan universal PCR Master Mix (Applied Biosystems) following manufacturer's instructions in a Step-One Plus Real-time PCR system (Applied Biosystems). To ensure reproducibility, a randomly selected 5% of the samples were re-genotyped and all the new results matched with genotypes initially detected.

**Table 1:** Autophagy polymorphisms analyzed in the study

*GENE*	*SNP ID*	*BASE CHANGE*	*PROTEIN CHANGE*	*CHR. LOCATION*	*ASSAY ID*^a^	*HWE*^b^
*ATG2B*	***rs3759601***	***4147C* > *G***	***p.Q1383E***	14:96,311,131	***C_9690160_20***	** > *0.05***
*ATG5*	***rs2245214***	***C* > *G***	***- (Intron)***	6:106,214,866	***C_3001905_20***	** > *0.05***
*ATG10*	***rs1864183***	***635C* > *T***	***p.T212M***	5:82,253,397	***C_11953871_10***	** > *0.05***
*ATG16L1*	***rs2241880***	***898A* > *G***	***p.T300A***	2:233,274,722	***C_9095577_20***	** > *0.05***
*NOD2*	***rs2066844***	***2104C* > *T***	***p.R702W***	16:50,712,015	C_11717468_20	** > *0.05***
*NOD2*	***rs2066845***	***2722C* > *G***	***p.R908G***	16:50,722,629	C_11717466_20	** > *0.05***

^a^All the assays were commercially

^b^*HWE* Hardy–Weinberg equilibrium in control group

### Statistical analysis

Since age, sex and race are important confounders of disease [23], patients were paired with control subjects with respect to sex to reduce variability and systematic differences due to background variables. Race was not an issue since our whole cohort was Caucasian. Nevertheless, the age of control group was skewed. As a control group, we have selected healthy subjects over 60 years old without any cancer or family history of cancer. Control group deviation from the Hardy–Weinberg equilibrium (HWE) was tested for each polymorphism using Pearson’s chi-squared test. The association between the different clinical (age, sex, location, type of resection, post-surgical treatment and overall survival) and molecular variables was analyzed by cross tabs, the Pearson’s *X*2 test and Fisher’s exact test when group size was < 5%. Odds ratios (ORs) and 95% confidence intervals (95% CIs) were estimated for each polymorphic variant using unconditional logistic regression analysis to evaluate the association with glioblastoma risk. Differences between groups were considered statistically significant where the *P*-value was < 0.05. 

For the survival analysis, overall survival time was stated as the survival rate in days from the diagnosis date to the time of death or the time of patient’s last check-up. Those patients who died during the follow up of the study were censored. In addition, patients who died within the next 14 days from surgery were excluded from analysis. The survival function was estimated using the Kaplan–Meier estimator. Differences across survival curves were compared by the log-rank method. All analyses were performed using SPSS software v.23.0 (IBM).

## Results

A total of 174 patients (53.6% males / 46.4% females) diagnosed of WHO IV grade glioblastoma were included in this study. The descriptive analysis of their clinicopathological features is summarized in Table 2. Samples from 264 individuals (58.7% males / 41.3% females) older than 60 and without personal or familial history of cancer were used as control population. All patients and controls were Spanish. The distribution of genotypes of all six polymorphisms in healthy subjects were in Hardy–Weinberg equilibrium (Table 1).

**Table 2:** Descriptive clinicopathological characteristics of patients included in the study

Tumor	Glioblastoma (*N* = 174)	
	**N**	**%**
**Sex**		
Male	89	53.6
Female	77	46.4
**Mean age, years [IQR]**	62.22 [27–79]	
**Tumor location**		
** Hemisphere**		
Right	83	50.3
Left	66	40
Other	16	9.7
** Lobe**		
Frontal	45	27.1
Temporal	47	28.3
Parietal	30	18.1
Occipital	8	4.8
Other	36	21.7
**Surgery**		
Total resection	97	71.9
Subtotal resection	38	28.1
**Post-surgery treatment**		
None	10	6.1
Radiotherapy	58	35.4
Radiotherapy + Chemotherapy	96	58.5
**Median survival, days [IC 95%]**	413.15 [7–4119]	

The distribution of allelic frequencies for each polymorphism studied and the susceptibility analysis to glioblastoma are shown in Table 3. No significant differences between groups in genotype distribution were found for *ATG16L1* rs2241880, *ATG5* rs2245214, *NOD2* rs2066845 polymorphisms. However, significant association with glioblastoma risk was found in *ATG2B* rs3759601, *ATG10* rs1864183 and *NOD2* rs2066844. In the case of *ATG2B* rs3759601, our study showed that homozygous GG genotype confers lower risk to develop glioblastoma in all codominant, recessive and dominant models (*p* = 0.000 OR = 0.284 (0.165–0.488); *p* = 0.001 OR = 0.645 (0.409–1.018) and *p* = 0.000 OR = 0.442 (0.266–0.735) respectively). *ATG10* rs1864183 was also unequally distributed between groups. Patients carrying TT genotype in this variant had higher risk to suffer from glioblastoma than those carrying CC in both codominant and recessive models (*p* = 0.030; OR** = **2,350 (1.282–4.307) and *p* = 0.018 OR = 1,863 (1.154–3.006) respectively). Finally, analysis of *NOD2* rs2066844 genotype distribution revealed that TT genotype was significantly associated with a higher risk of developing glioblastoma when compared with the most frequent genotype in both codominant and dominant models (*p* = 0.018 OR = 4,948 (0.547–44,79) and *p* = 0.006 OR = 2,193 (1.241–3.877)).

**Table 3:** Comparative results of genotypic frequencies of selected polymorphisms in cases and controls and the association with glioblastoma risk. Significant *P*-values are represented in bold

SNP	Inheritance model	Genotype	Patients N (%)	Controls N (%)	*p*-value	OR (IC 95%)
***ATG2B rs3759601***	Codominant	CC	55 (31.6%)	39 (14.8%)	**0.000**	1
		CG	73 (42%)	115 (43.6%)		0.433 (0.259–0.721)
		GG	46 (26.4%)	110 (41.7%)		0.284 (0.165–0.488)
	Recessive	CC + CG	18 (73.6%)	154 (78.3%)	**0.001**	1
		GG	46 (26.4%)	110 (41.7%)	/	0.645 (0.409–1.018)
	Dominant	CC	55 (31.6%)	39 (14.8%)	**0.000**	1
		CG + GG	119 (68.4%)	225 (85.2%)	/	0.442 (0.266–0.735)
***ATG5 rs2245214***	Codominant	CC	67 (38.5%)	106 (40.2%)	0.926	
		CG	85 (38.9%)	127 (48.1%)		
		GG	22 (12.6%)	31 (11.7%)		
	Recessive	CC + CG	152 (87.4%)	223 (88.3%)	0.777	
		GG	22 (12.6%)	31 (11.7%)	/	
	Dominant	CC	67 (38.5%)	106 (40.2%)	0.730	
		CG + GG	107 (61.5%)	158 (59.8%)	/	
***ATG10 rs1864183***	Codominant	CC	32 (18.4%)	68 (25.8%)	**0.030**	1
		CT	96 (55.2%)	151 (57.2%)		1.379 (0.831–2.290)
		TT	46 (26.4%)	45 (17%)		2.350 (1.282–4.307)
	Recessive	CC + CT	128 (73.6%)	219 (83%)	**0.018**	1
		TT	46 (26.4%)	45 (17%)	/	1.863 (1.154–3.006)
	Dominant	CC	32 (18.4%)	68 (25.8%)	0.072	
		CT + TT	142 (81.6%)	196 (74.2%)	/	
***ATG16L1 rs2241880***	Codominant	GG	45 (25.9%)	63 (23.9%)	0.784	
		GA	92 (52.9%)	138 (52.3%)		
		AA	37 (21.3%)	63 (23.9%)		
	Recessive	GG + GA	137 (78.7%)	201 (76.1%)	0.526	
		AA	37 (21.3%)	63 (23.9%)	/	
	Dominant	GG	45 (25.9%)	63 (23.9%)	0.635	
		GA + AA	129 (74.1%)	201(76.1%)	/	
***NOD2 rs2066844***	Codominant	CC	135 (77.6%)	167 (88.4%)	**0.018**	1
		CT	35 (20.1%)	21 (11.1%)		2.062 (1.147–3.707)
		TT	4 (2.3%)	1 (0.5%)		4.948 (0.547–44.79)
	Recessive	CC + CT	170 (97.7%)	188 (99.5%)	0.148	
		TT	4 (2.3%)	1 (0.5%)	/	
	Dominant	CC	135 (77.6%)	167 (88.4%)	**0.006**	1
		CT + TT	39 (22.4%)	22 (11.6%)	/	2.193 (1.241–3.877)
***NOD2 rs2066845***	Codominant	GG	168 (96.6%)	100 (99%)	0.212	
		GC	6 (3.4%)	1 (1%)		
		CC	-	-		
	Recessive	GG + GC	174 (100%)	101 (100%)	-	
		CC	-	-	/	
	Dominant	GG	168 (96.6%)	100 (99%)	0.212	
		GC + CC	6 (3.4%)	1 (1%)	/	

The distribution of allelic frequencies for selected polymorphisms confirmed statistically significant differences between cases and control subjects for *ATG2B* rs3759601, *ATG10* rs1864183 and *NOD2* rs2066844 (Table 4). Being a carrier of the G allele of *ATG2B* rs3759601 polymorphism confers a decreased risk of developing glioblastoma (*p* = 0.001 OR = 0.519 (0.395–0.684)). On the contrary, carrying the T allele for both *ATG10* rs1864183 and *NOD2* rs2066844 polymorphisms confers higher risk of developing glioblastoma *p* = 0.001 OR = 0.399 (1.066–1.836) and *p* = 0.001 OR = 2.173 (1.282–3.693) respectively).

**Table 4:** Comparative results of genotypic frequencies of selected polymorphisms in cases and controls and the association with glioblastoma risk. Significant *P*-values are represented in bold

SNP	Allele	Cases N (%)	Controls N (%)	*p*-value	OR (IC 95%)
***ATG2B rs3759601***	C	183 (52.6%)	193 (36.6%)	**0.000**	1
	G	165 (47.4%)	335 (63.4%)	/	0.519 (0.395–0.684)
***ATG5 rs2245214***	C	219 (62.9%)	339 (64.2%)	0.720	
	G	129 (37.1%)	189 (35.8%)	/	
***ATG10 rs1864183***	C	160 (46%)	287 (54.4%)	**0.016**	1
	T	188 (54%)	241 (45.6%)	/	0.399 (1.066–1.836)
***ATG16L1 rs2241880***	G	128 (52.3%)	264(50%)	0.505	
	A	166 (47.7%)	264 (50%)	/	
***NOD2 rs2066844***	C	305 (87.6%)	355 (93.9%)	**0.003**	1
	T	43 (12.4%)	23 (6.1%)	/	2.173 (1.282–3.693)
***NOD2 rs2066845***	G	342 (98.3%)	201 (99.5%)	0.21	
	C	6 (1.7%)	1 (0.5%)	/	

**Fig. 1:**
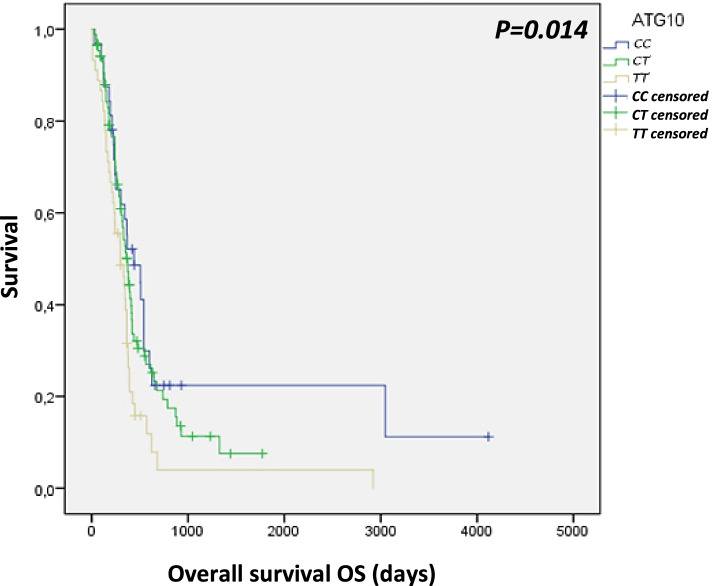
Kaplan–Meier survival curve for ATG10 rs1864183 in patients of our study cohort

The relationship between the different clinical features listed in glioblastoma patients and candidate gene polymorphisms was also investigated (Additional file Table 1). *ATG2B* rs3759601 distribution showed that carriers of the genotype GG appeared to have higher probability of administering a gross total resection during surgery and a complete postsurgical management with radiotherapy and temozolomide (χ^2^ = 18.122; *P* = 0.001 y χ^2^ = 6.069; *P* = 0.048 respectively). GC genotype of *ATG5* rs2245214 also exhibited higher frequencies in patients with complete postsurgical treatment (χ^2^ = 9.530; *P* = 0.049). *NOD2* rs2066844 genotype distribution revealed that TT genotype was more frequent in males (χ^2^ = 8.796; *P* = 0.012) and in younger patients (< 63 years old) (χ^2^ = 6.818; *P* = 0.033). In addition, Kaplan–Meier analysis showed that patients carrying the TT genotype for *ATG10* rs1864183 presented shorter survivals, suggesting that *ATG10* rs1864183 might be related to the prognosis of glioblastoma patients (Fig. 1).

## Discussion

Autophagy is an essential process in eukaryotic cells that has proved to be altered in different diseases including cancer. The role of this mechanism in glioblastoma is still controversial due to its dual role in promoting both tumor cell death and cell survival. Nevertheless, several evidences suggest that autophagy may be involved in the initiation, promotion and progression of this tumor [7]. With this study, we aim to unveil the possible association of several selected polymorphisms in genes involved in autophagy and the susceptibility to develop glioblastoma. Five of these polymorphisms were exonic missense changes (*ATG2B* rs3759601, *ATG16L1* rs2241880, *ATG10* rs1864183, *NOD2* rs2066844 and rs2066845) while one (*ATG5* rs2245214) was an intronic mutation located in a recognition site for SRp40 splicing factor.

Atg2B is a crucial protein involved in autophagosome formation and regulation of size and distribution of lipid droplets [24]. *ATG2B* has been described as a predisposition gene in familial myeloproliferative neoplasms (predisposition)[25, 26] and has been associated to colorectal cancer [27]. Furthermore, rare variants in this gene have been correlated with survival in ovarian cancer [28]. In this study, we have assessed the association between glioblastoma development and polymorphism *ATG2B* rs3759601. This C > G transversion present in exon 5 produces a glutamine-to-glutamic acid change in position 1383 (p.Gln1383Glu) and has been associated to higher risk to suffer pharyngeal cancer [29]. Ours results showed that patients carrying GG genotype had higher probability of receiving a gross total resection since, in most cases, tumors were located in more accessible sites, and they received complete postsurgical treatment including chemotherapy and radiotherapy. However, GG genotype was not correlated with longer survival times. Furthermore, GG and GC genotypes decrease glioblastoma risk and that carrying the G allele might confer protection against the disease.

*ATG5* codes for an essential protein for autophagy vesicle formation by participating in the Atg5-Atg12-Atg16L1 conjugation complex [30]. Polymorphism *ATG5* rs2245214 has been previously studied in patients with head and neck squamous cell carcinoma [29], non-medullary thyroid cancer [31], Paget disease of bone [32], tuberculosis [33] and lupus erythematous [34] with heterogeneous results. In this work, we did not find any difference in the polymorphism distribution in patients suffering from glioblastoma and control cases. This might be due to the fact that this variant affects intronic region 6 of *ATG5* gene and this change might not disturb protein function, resulting in no changes in autophagy.

Atg10 is an essential E2-like enzyme that mediates the formation of Atg12-Atg5 conjugate [35]. Increased expression of this protein has been associated with lymphovascular invasion and lymph node metastasis of colorectal cancer [36]. Nonsynonymous *ATG10* variant rs1864183 encodes a threonine-to-methionine change at codon 212. It has been predicted to be located at exonic splicing enhancers (ESEs) and has been proposed to lead to the catalytic change of the protein, causing a dysregulation of autophagosome formation and, eventually, resulting in altered risk of breast cancer [37]. This mutation has been associated with higher risk to develop laryngeal cancer [29] and hepatocellular carcinoma [38]. Analysis of glioblastoma patients showed a correlation between allele T and its related genotypes and a higher risk to develop glioblastoma. It has been reported that down-regulation of ATG genes decreases autophagy and accelerates tumor progression [39]. We could hypothesize that variant rs1864183 might decrease autophagy and, thus, be related to higher risk to glioblastoma. Interestingly, we found that TT genotype is associated to shorter overall survival times in glioblastoma patients, suggesting that *ATG10* rs1864183 might be related to the prognosis of glioblastoma patients. In fact, this polymorphism has already been associated with overall survival in non-small cell lung cancer patients treated with platinum-based chemotherapy [40]. Cao et al. suggested that Atg10 protein might serve as a prognostic biomarker in gastric cancer [41]. These findings insinuate that *ATG10* might be a potential predictor for clinical outcomes in cancer.

Atg16L1 is an indispensable factor for autophagy vesicle formation by being part of the Atg5-Atg12-Atg16L1 conjugation complex [18]. *ATG16L1* rs2241880 produces a A > G transition that encodes a protein change in position 300 (p.T300A). It has been reported that variant T300A enhances ATG16L1 degradation by caspase 3, leading to a defective autophagy and higher inflammation [42]. This variant has been associated to increase susceptibility to develop Crohn’s disease [43, 44], Paget disease of bone [32], oral cavity squamous cell carcinoma [29], brain metastases in patients with non-small cell lung cancer and gastric cancer [45]. Surprisingly, no correlation was found in the case of hepatocellular carcinoma [38]. In the current study, we could not find any correlation between *ATG16L1* rs2241880 distribution and the susceptibility to develop glioblastoma.

Nod2 plays a relevant role in innate immunity by detecting intracellular bacteria and activating the nuclear factor-kappaB pathway [46]. Moreover, Nod2 role has been recently expanded as it has been reported as a nucleating factor for the initiation of bacteria-induced autophagy by recruiting Atg16L1 [47]. In the present work, we have assessed the impact of *NOD2* rs2066844 and rs2066845 variants in glioblastoma development. These mutations are located in C-terminal region and generate missense changes in the protein (R702W and G908R, respectively). Both *NOD2* rs2066844 and rs2066845 polymorphisms have been associated to higher risk to Crohn’s disease [48] and several types of cancer [49]. However, we did not find any significant correlation in *NOD2* rs2066845 distribution and glioblastoma susceptibility. Nevertheless, we found that genotype CT and TT in *NOD2* rs2066844 confers 2.0- and 4.9-times higher risk to develop this tumor, respectively. T allele has been reported as unable to respond to bacterial muramyldipeptide and activate NF-KB [49]. We could infer that this allele results in defective autophagy, leading to an increased risk to develop glioblastoma. Additionally, we found that genotype TT is more frequent in males as well as < 63-year-old patients. This age-dependent correlation was also observed in Portuguese [50] and German [51] populations. This fact also correlates with epidemiology data supporting that glioblastoma incidence rates are higher in males than in females when patients are younger than 63 years-old [52]. It could be hypothesized that defective autophagy due to *NOD2* rs2066844 TT genotype might contribute to early manifestation of glioblastoma.

Our study had some limitations. First, the sample size of the present case–control study (174 subjects) is not too large due to the difficulty of enrolling an adequate number of patients and, thus, biases might exist. Therefore, additional studies with a larger sample size are required to validate our findings and confirm that they are applicable to the general population suffering from glioblastoma. Second, doubts may arise regarding the reliability of the controls selected in our study. However, despite the fact that patients’ recruitment was carried out in different hospitals, all of them were Caucasian with similar educational level that shared socio-demographic characteristics, and none belonged to ethnic minority groups. In addition, previous diseases and health conditions, such as obesity, physical activity, smokers or potential pharmacological treatment, were considered to guaranty that control subjects represented selected patients and, therefore, assure their validity.

Finally, further investigation will be key to evaluate additional associations between autophagy-related genes and glioblastoma development. Interestingly, two recent reports have pointed out a correlation between high ATG gene expression signatures and worst outcomes in glioblastoma patients, particularly with mesenchymal subtype [53, 54]. Therefore, it will be essential to assess in future experiments if the analyzed autophagy-related genes could be used to construct a high-risk signature that might act as prognostic factor for glioblastoma.

## Conclusion

In conclusion, the present study provides evidence of the potential role of several polymorphisms in autophagy genes as genetic predisposing factors in glioblastoma development. To the best of our knowledge, this is the first susceptibility study analyzing the association of presumed functional variants of ATGs and NOD2 with glioblastoma risk taking into account clinical features of the cohort. In addition, we describe for the first time *ATG10* rs1864183 as a putative promising prognosis factor for this tumor. Our results further support the belief that autophagy contributes to carcinogenesis in general, and glioblastoma development in particular. Further studies in different and larger sample sizes and functional analysis of these polymorphisms are required to validate our findings.

## Supplementary Information

**Additional file 1: ST1.** Clinicopathological features associationwith selected polymorphisms distribution in glioblastoma patients.

**Additional file 2.** Raw data.

## Data Availability

All data generated and analysed during this study are included in this published article and its supplementary information files.

## Abbreviations

GBM: Glioblastoma
TMZ: Temozolomide
WHO: World Health Organization
