# Supplementary material for: Polymorphisms in autophagy genes are genetic susceptibility factors in glioblastoma development
Source: BMC Cancer. 2022 Feb 5;22:146. doi: 10.1186/s12885-022-09214-y (PMC8818195; doi:10.1186/s12885-022-09214-y)
Supplement: Supplementary file 1 — Additional file 1: ST1. Clinicopathological features associationwith selected polymorphisms distribution in glioblastoma patients. [file 12885_2022_9214_MOESM1_ESM.docx]

Supplemental table 1: Clinicopathological features association with selected polymorphisms distribution in glioblastoma patients. Significant P-values are represented in bold.

| ATG2B rs3759601 | | CC | CG | GG | Chi-square | P-value |
| --- | --- | --- | --- | --- | --- | --- |
| Sex | **Female** | 21 | 34 | 22 | 1.525 | .466 |
|  | **Male** | 32 | 33 | 24 |  |  |
| Age | **<63** | 23 | 26 | 23 | 1.242 | .538 |
|  | **>63** | 30 | 40 | 23 |  |  |
| Treatment | **R+Q** | 21 | 39 | 36 | 18.122 | **.001** |
|  | **R** | 28 | 24 | 6 |  |  |
|  | **No** | 4 | 4 | 2 |  |  |
| Hemisphere | **Left** | 23 | 23 | 20 | 7.221 | .125 |
|  | **Right** | 21 | 38 | 24 |  |  |
|  | **Other** | 9 | 5 | 2 |  |  |
| Location | **Frontal** | 14 | 18 | 13 | 3.273 | .916 |
|  | **Temporal** | 18 | 20 | 9 |  |  |
|  | **Parietal** | 8 | 12 | 10 |  |  |
|  | **Occipital** | 3 | 3 | 2 |  |  |
|  | **Other** | 10 | 14 | 12 |  |  |
| Resection | **Total** | 39 | 32 | 26 | 6.069 | **.048** |
|  | **Subtotal** | 14 | 20 | 4 |  |  |
| Survival | | | | | 4.538 | .103 |
| ATG5 rs2245214 | | **CC** | **CG** | **GG** | **Chi-square** | **P-value** |
| Sex | **Female** | 29 | 39 | 9 | .236 | .889 |
|  | **Male** | 35 | 42 | 12 |  |  |
| Age | **<63** | 24 | 38 | 10 | 1.601 | .449 |
|  | **>63** | 40 | 42 | 11 |  |  |
| Treatment | **R+Q** | 42 | 47 | 7 | 9.530 | **.049** |
|  | **R** | 16 | 29 | 13 |  |  |
|  | **No** | 5 | 4 | 1 |  |  |
| Hemisphere | **Left** | 28 | 33 | 5 | 6.531 | .163 |
|  | **Right** | 27 | 41 | 15 |  |  |
|  | **Other** | 9 | 6 | 1 |  |  |
| Location | **Frontal** | 19 | 19 | 7 | 9.365 | .312 |
|  | **Temporal** | 17 | 24 | 6 |  |  |
|  | **Parietal** | 8 | 19 | 3 |  |  |
|  | **Occipital** | 1 | 5 | 2 |  |  |
|  | **Other** | 19 | 14 | 3 |  |  |
| Resection | **Total** | 34 | 51 | 12 | 1.068 | .586 |
|  | **Subtotal** | 14 | 17 | 7 |  |  |
| Survival | | | | | .043 | .979 |
| ATG10 rs1864183 | | **CC** | **CT** | **TT** | **Chi-square** | **P-value** |
| Sex | **Female** | 17 | 39 | 21 | .821 | .663 |
|  | **Male** | 15 | 50 | 24 |  |  |
| Age | **<63** | 17 | 37 | 18 | 1.504 | .471 |
|  | **>63** | 15 | 51 | 27 |  |  |
| Treatment | **R+Q** | 18 | 49 | 29 | 6.125 | .190 |
|  | **R** | 12 | 36 | 10 |  |  |
|  | **No** | 1 | 4 | 5 |  |  |
| Hemisphere | **Left** | 13 | 35 | 18 | 4.651 | .325 |
|  | **Right** | 19 | 43 | 21 |  |  |
|  | **Other** | 0 | 10 | 6 |  |  |
| Location | **Frontal** | 8 | 24 | 13 | 4.713 | .788 |
|  | **Temporal** | 8 | 25 | 14 |  |  |
|  | **Parietal** | 8 | 15 | 7 |  |  |
|  | **Occipital** | 2 | 6 | 0 |  |  |
|  | **Other** | 6 | 19 | 11 |  |  |
| Resection | **Total** | 17 | 59 | 21 | 2.313 | .315 |
|  | **Subtotal** | 6 | 19 | 13 |  |  |
| Survival | | | | | 8.592 | **.014** |
| ATG16L1 rs2241880 | | **GG** | **GA** | **AA** | **Chi-square** | **P-value** |
| Sex | **Female** | 23 | 38 | 16 | 2.097 | .351 |
|  | **Male** | 18 | 51 | 20 |  |  |
| Age | **<63** | 18 | 37 | 17 | .280 | .869 |
|  | **>63** | 23 | 51 | 19 |  |  |
| Treatment | **R+Q** | 32 | 47 | 17 | 8.934 | .063 |
|  | **R** | 8 | 35 | 15 |  |  |
|  | **No** | 1 | 6 | 3 |  |  |
| Hemisphere | **Left** | 16 | 38 | 12 | 2.574 | .631 |
|  | **Right** | 20 | 41 | 22 |  |  |
|  | **Other** | 4 | 10 | 2 |  |  |
| Location | **Frontal** | 9 | 28 | 8 | 10.151 | .255 |
|  | **Temporal** | 13 | 22 | 12 |  |  |
|  | **Parietal** | 6 | 13 | 11 |  |  |
|  | **Occipital** | 2 | 6 | 0 |  |  |
|  | **Other** | 11 | 20 | 5 |  |  |
| Resection | **Total** | 17 | 53 | 27 | .241 | .887 |
|  | **Subtotal** | 7 | 22 | 9 |  |  |
| Survival | | | | | .438 | .803 |
| NOD2 rs2066844 | | **CC** | **CT** | **TT** | **Chi-square** | **P-value** |
| Sex | **Female** | 56 | 21 | 0 | 8.796 | **.012** |
|  | **Male** | 74 | 11 | 4 |  |  |
| Age | **<63** | 61 | 8 | 3 | 6.818 | **.033** |
|  | **>63** | 68 | 24 | 1 |  |  |
| Treatment | **R+Q** | 73 | 22 | 1 | 15.604 | .112 |
|  | **R** | 48 | 7 | 3 |  |  |
|  | **No** | 7 | 3 | 0 |  |  |
| Hemisphere | **Left** | 53 | 11 | 2 | 26.139 | .052 |
|  | **Right** | 69 | 12 | 2 |  |  |
|  | **Other** | 7 | 9 | 0 |  |  |
| Location | **Frontal** | 36 | 6 | 3 | 21.383 | .164 |
|  | **Temporal** | 40 | 6 | 1 |  |  |
|  | **Parietal** | 22 | 8 | 0 |  |  |
|  | **Occipital** | 6 | 2 | 0 |  |  |
|  | **Other** | 26 | 10 | 0 |  |  |
| Resection | **Total** | 73 | 21 | 3 | 12.501 | .130 |
|  | **Subtotal** | 28 | 9 | 1 |  |  |
| Survival | | | | | 1.303 | .521 |
| NOD2 rs2066845 | | **GG** | **GC** | **Chi-square** | | **P-value** |
| Sex | **Female** | 74 | 3 | .384 | | .535 |
|  | **Male** | 87 | 2 |  |  |  |
| Age | **<63** | 70 | 2 | .028 | | .868 |
|  | **>63** | 90 | 3 |  |  |  |
| Treatment | **R+Q** | 92 | 4 | 5.046 | | .410 |
|  | **R** | 58 | 0 |  |  |  |
|  | **No** | 9 | 1 |  |  |  |
| Hemisphere | **Left** | 65 | 1 | 1.854 | | .985 |
|  | **Right** | 80 | 3 |  |  |  |
|  | **Other** | 15 | 1 |  |  |  |
| Location | **Frontal** | 43 | 2 | 3.744 | | .879 |
|  | **Temporal** | 47 | 0 |  |  |  |
|  | **Parietal** | 29 | 1 |  |  |  |
|  | **Occipital** | 8 | 0 |  |  |  |
|  | **Other** | 34 | 2 |  |  |  |
| Resection | **Total** | 94 | 3 | .247 | | .993 |
|  | **Subtotal** | 37 | 1 |  |  |  |
| Survival | | | | 1.050 | | .306 |
